# Aggravated Case of Neuralgia (Neuritis?) of Fifth Nerve

**Published:** 1895-09

**Authors:** Joseph T. O’Connor

**Affiliations:** New York


### AGGRAVATED CASE OF NEURALGIA (NEURITIS?) OF FIFTH NERVE. RELIEVED BY NATRUM-SULPHURICUM.

JOSEPH T. O'CONNOR, M. D., NEW YORK.

On January 4th, 1893, Rev. Mr. —— was referred to me by Dr. S. F. Wilcox for my opinion as to the advisability of evulsion of the left fifth nerve for a frightfully severe neuralgia. The history was as follows: Some ten years ago he had a second molar tooth extracted from upper jaw, right side. Bleeding continued for eight days; there was some slight pain on touching the vacant space and on washing the face. He had a bad attack of neuralgia in that side in about three months; it lasted a short while. This was repeated, and the attacks gradually grew longer in duration and more frequent. Two years ago he underwent the operation of resection of the maxillary branch of the nerve at the foramen in the malar bone, and experienced relief from pain during the following summer. The trouble, however, returned in the autumn or early winter, and now he has no relief from pain. At present the pain is worse after midnight. Any movement of jaws or tongue will aggravate existing pain or bring on a frightful paroxysm. A drink of cold water aggravates, while hot water sometimes relieves. Is somewhat easier by lying down but is intensely restless; cannot lie still at night. There is no thirst; feet and hands always cold. Bowels regular, but has piles, which get worse when the neuralgia is worse. He could not open his mouth to show me his tongue, and it was extremly difficult to understand his speech. Upon asking how he managed to preach he said that he did but little of that, and that after he got excited he could open his mouth somewhat and so make necessary announcements to his congregation or preach a short sermon.

Arsenicum$^{30}$ was prescribed. In ten days he returned, saying that his nights were not so bad but that there was no other essential improvement. I could now examine his tongue, and found it coated in its posterior two-thirds with a thick yellowish-brown layer. As this colored coating of the tongue is given by Schüssler as a characteristic for Natrum-sulphuricum, I was anxious to get some more and better indications for that remedy, and upon further inquiry I found that the trouble began when he was living in a very damp house, the walls of which were frequently moist, and that the earlier attacks always came after he had been down in the cellar. On this indication I gave Natrum-sulph.$^{6}$, to be taken in water, with instructions to cease taking it as soon as any improvement was noted. The result was marvelous; he got practically well, and in the course of a few months took a parish in another part of the country. Had not the operation been performed on the nerve I feel sure that the cure would have been absolute before he left.

My chief object in recording this case is to urge upon the practitioners of Homœopathy to seek always for the cause of the trouble in chronic diseases and then to prescribe according to the causal indication where such exists in the materia medica. This is the only short cut that I know of in selecting a remedy.

